# The Prevalence of Cardiovascular–Kidney–Metabolic Syndrome: A Review of Published Estimates and New Findings from BRFSS Surveys

**DOI:** 10.3390/cardiovascmed29010005

**Published:** 2026-02-03

**Authors:** Steven S. Coughlin, Nikul Parikh, Ashley Oh, Biplab Datta, Marlo Vernon, Jennifer Sullivan

**Affiliations:** 1 Department of Biostatistics, Data Science and Epidemiology, School of Public Health, Augusta University, 1120 15th Street, Augusta, GA 30912, USA; 2 Institute of Public and Preventive Health, School of Public Health, Augusta University, Augusta, GA 30912, USA; 3 Medical College of Georgia, Augusta University, Augusta, GA 30912, USA; 4 Department of Health Management, Economics and Policy, School of Public Health, Augusta University, Augusta, GA 30912, USA; 5 Georgia Prevention Institute, Augusta University, Augusta, GA 30912, USA; 6 Department of Obstetrics and Gynecology, Medical College of Georgia, Augusta University, Augusta, GA 30912, USA; 7 Department of Physiology, Medical College of Georgia, Augusta University, Augusta, GA 30912, USA

**Keywords:** African Americans, cardiovascular disease, chronic kidney disease, diabetes, obesity, women

## Abstract

Because CKMS was only proposed by the American Heart Association in 2023, there has been a paucity of information about the distribution and determinants of the syndrome across population groups. We reviewed published studies of the prevalence of CKMS in the U.S. and other countries and obtained new estimates of the prevalence of this syndrome among U.S. adults by birth decade and sociodemographic attributes using 2019, 2021, and 2023 Behavioral Risk Factor Surveillance System (BRFSS) data. The results of this study indicate that CKMS is widespread in the general U.S. population, especially among older cohorts born before 1940 and during the 1940s, 1950s, and 1960s. Except for the three younger cohorts, born in the 1980s, 1990s, and 2000 or later, the prevalence of CKMS stage 4 was significantly higher among males than in females. Among those born between the 1950s and 1990s, the prevalence was significantly higher among non-Hispanic Blacks compared to their non-Hispanic white counterparts. Across all birth decades, prevalence of CKMS stage 4 was generally higher among those without a college degree, from a low-income household, and residing in rural areas. These prevalence rate estimates will further our understanding of the burden and unique needs of different population groups in improving cardiovascular–kidney–metabolic health across the life course.

## 1. Introduction

Cardiovascular–Kidney–Metabolic Syndrome (CKMS) is an emerging public health concern characterized by a progressive decline in kidney function that cannot be attributed solely to traditional causes [1]. It describes a cluster of inter-related conditions, including cardiovascular disease (CVD), chronic kidney disease (CKD), and metabolic disorders such as obesity and diabetes, that are associated with increased morbidity and mortality [1]. These inter-related conditions are among the leading causes of death in the United States and other countries [2–4]. Previous studies have examined the prevalence of CKMS in certain populations in the U.S. and other countries [3].

Because CKMS was recently proposed by the American Heart Association in 2023 [1], there has been a paucity of information about the distribution and determinants of the syndrome across different population groups. Additionally, CKMS can have a distinct impact depending on gender [5]. For example, CKMS can adversely affect reproductive health in women, contributing to complications such as pre-eclampsia, preterm birth, and increased maternal morbidity [6], differentially impacting females across certain age groups. Despite these significant risks, CKMS remains under-recognized and under-researched in populations stratified by age, sex and gender, race and ethnicity, and socioeconomic attributes, underscoring the need for targeted screening, prevention, and treatment strategies.

Although published estimates provide some information about the prevalence of CKMS by sex and by race, estimates have been lacking for proper stratification by age, which is a biological risk factor for both CVD and CKD.

To further understand the burden of CKMS, we obtained new estimates of the prevalence of this syndrome among U.S. adults using 2019, 2021, and 2023 Behavioral Risk Factor Surveillance System data, stratifying observations by birth decade and sociodemographic characteristics. We also conducted a review of published studies of the prevalence of CKMS in the U.S. and other countries. Our analyses add to the literature by providing estimates of the prevalence of CKMS among subgroups of U.S. adults by demographic and socioeconomic characteristics across different birth cohorts.

## 2. Methods

### 2.1. Literature Review

A literature review was completed based upon bibliographic searches in PubMed and CINAHL and relevant search terms. Articles published in English from 2023 through April 1, 2025 were identified using the following MeSH search terms and Boolean algebra commands: cardiovascular-kidney-metabolic syndrome AND prevalence. The searches were not limited to words appearing in the title of an article nor to studies in a particular country or geographic region of the world. The references of review articles were also reviewed. Information obtained from bibliographic searches (title and topic of article, information in abstract, study design, and keywords) was used to determine whether to retain each article identified in this way. Only studies written in English that examined the prevalence of CKMS were eligible for inclusion. A total of 66 articles were identified in the bibliographic searches. Of these, 9 were eligible for inclusion. No additional articles were identified in the CINAHL search.

### 2.2. Behavioral Risk Factor Surveillance System

BRFSS is a cross-sectional telephone-based survey of U.S. residents aged 18 years and older, which collects information about health-related risk factors, chronic health conditions, and use of preventive services. We used the three most recent waves of the BRFSS, 2019, 2021, and 2023, which contain necessary information about conditions (e.g., hypertension, diabetes, etc.) for determining CKMS stages.

The following self-reported health conditions were assessed to determine the stages of CKMS: heart attack (myocardial infarction), coronary heart disease (CHD), stroke, prediabetes, diabetes, pre-hypertension, hypertension, high cholesterol, chronic kidney disease, and overweight/obesity. Participants were asked if they had ever been told by a doctor or a health professional that they had one of these chronic conditions. Those who responded positively to the respective questions were identified as having that chronic condition. Respondents’ body mass index (BMI) values, based on self-reported height and weight, were used to assess overweight and obesity conditions.

CKMS stage 0 was determined as BMI < 25 kg/m^2^ and not having any of the following conditions: pre-diabetes, diabetes, hypertension, pre-hypertension, high cholesterol, CKD, and CVD. CKMS stage 1 was determined as BMI ≥ 25 kg/m^2^ or having pre-diabetes, and not having any of the following conditions: diabetes, hypertension, high cholesterol, CKD, and CVD. CKMS stage 2 was determined as not having CVD and having any of the following conditions: high cholesterol, hypertension, diabetes, metabolic syndrome, or CKD. A key component of CKMS stage 3 is subclinical CVD, for which we did not have information in the BRFSS data. As such, following practice in the extant literature [7], we consolidated CKMS stages 2 and 3 in one single group. In addition to the components of CKMS stage 2, stage 3 included having any of the following conditions: BMI ≥ 25 kg/m^2^, or pre-diabetes. Lastly, CKMS stage 4 was determined as having CVD and any of the following conditions: BMI ≥ 25 kg/m^2^, pre-diabetes, diabetes, hypertension, high cholesterol, metabolic syndrome, or CKD. A detailed description linking the clinical definition of CKMS components [8] and their proxies used in the BRFSS data is presented in Supplemental Materials Table S1. Metabolic syndrome was defined as having three or more of the following conditions: BMI ≥ 25 kg/m^2^, diabetes, hypertension, and high cholesterol [9]. Note that the BRFSS does not provide information about abdominal obesity, lipid profile, systolic and diastolic blood pressure levels, blood glucose levels, HbA1c, non-clinical and clinical determination of CVD, and risk-levels of CKD. As such, we were unable to incorporate these factors in determining the stages of CKMS.

We first examined the prevalence of CKMS stages by the following birth decades: before 1940 (≤1939), during the 1940s (1940–1949), 1950s (1950–1959), 1960s (1960–1969), 1970s (1970–1979), 1980s (1980–1989), 1990s (1990–1999), and after 1999 (≥2000). Birth years were estimated by subtracting respondents’ age at the time of the interview from the year of the interview. Birth decades were represented by age groups as follows: ≤1939: 80+ years; 1940s: 70–80 years; 1950s: 60–74 years; 1960s: 50–64 years; 1970s: 40–54 years; 1980s: 30–44 years; 1990s: 20–34 years; and ≥2000: 18–24 years.

Next, we examined the prevalence rates by demographic factors including sex and race and ethnicity across the birth decades. Lastly, we examined the prevalence rates by socioeconomic conditions including educational attainment, income, and urban/rural residence across birth decades. There were three education groups—high school or less, some college, and college degree. The three income groups were low income (<200% of the Federal Poverty Line [FPL]), middle income (200% to <400% of the FPL), and high income (≥400% of the FPL). Income categories reported in the BRFSS data were converted to continuous income using uniform distribution method [10] and Federal Poverty Guidelines of respective years were used to obtain FPL estimates. Urban counties comprising Metropolitan Status Codes 1 to 5 (based on the 2013 NCHS Urban-Rural Classification Scheme for Counties) were considered as urban and rural counties defined by Metropolitan Status Code 6 were considered as rural areas of residence.

Prevalence rates (in percentage) and 95% confidence intervals were estimated using the complex survey waves of the BRFSS, adjusted for the pooled structure of the data. Adjusted weights were calculated following instructions provided in the BRFSS website for combining multiple years of BRFSS data. Observations for which data were missing were excluded from analysis.

For assessing differences, multinomial logistic regressions for CKMS stages were estimated. CKMS stage 0 was considered as the base category. Unadjusted relative risk ratios for demographic and socioeconomic characteristics were obtained and presented in the Supplemental Material tables. For each characteristic (i.e., sex, race and ethnicity, education, income, and urban/rural residence), separate regressions by birth decades were estimated. As such, differences across demographic/socioeconomic attributes (e.g., male vs. female) were reported within birth decades. For assessing overall differences across birth decades, a separate regression was estimated using the full sample with adjustments made for demographic and socioeconomic characteristics. Statistical analyses were conducted in Stata 18.0 software.

## 3. Results

Because CKMS is a relatively new syndrome, prevalence estimates have only recently become available. Using the National Health and Nutrition Examination Survey (NHANES) data from 2011 to 2020, Aggarwal et al. [3] examined the prevalence of CKMS stages among U.S. adults (Table 1). Between 2011 and 2020, 10.6% of U.S. adults met criteria for stage 0, 25.9% for stage 1, 49.0% for stage 2, 5.4% for stage 3, and 9.2% for stage 4. The prevalence of each stage did not significantly change over the study period (*p* for trend > 0.05 for each stage). Adults 65 years or older were more likely to have advanced CKMS stages than those aged 45 to 64 years (55.3% vs. 10.7%, *p* < 0.001) or 20 to 44 years (53.3% vs. 2.1%, *p* < 0.001) (10). Compared with women, men were more likely to have advanced stages (16.9% vs. 12.4%, adjusted prevalence ratio [PR] = 1.36, 95% CI 1.24–1.49, *p* < 0.001), and Black adults were significantly more likely than White adults to have advanced-stage CKMS (18.9% vs. 13.8%, adjusted PR = 1.38, 95% CI 1.24–1.55, *p* < 0.001). Additional results were obtained by Zhu et al. [11] in an analysis of NHANES data from 1999 to 2018. The age-adjusted prevalence of CKMS stage 0 to 4 was 13.6%, 29.9%, 43.7%, 4.7%, and 8.1%, respectively. The highest proportion of stage 4 CKMS was among adults aged ≥65–79 years, males, and non-Hispanic Black individuals.

Minhas et al. [12] analyzed NHANES data from 2011 to 2018 in adults aged ≥20 years. Pregnant women were excluded. Among those aged 20 to 44, 45 to 64, and ≥65 years, stage 0 CKM was present in 17.35%, 5.45%, and 1.80%, respectively, and stages 1–3 were present in 80.94%, 85.95%, and 72.03%, respectively. The highest proportion of stage 4 was among adults aged ≥65 years, males, and non-Hispanic white and non-Hispanic Black individuals. Additional results were obtained by Kim et al. [13] using NHANES data from 2011 to 2018. The age-adjusted prevalence of stages 0–4 were 11.2%, 28.1%, 47.4%, 5.3%, and 8.1%, respectively. The highest proportion of stage 4 was among adults aged ≥60 years, males, and non-Hispanic Black individuals.

Ji et al. [14] analyzed NHANES data from 1988 to 2018 collected from U.S. adults who were under surveillance for mortality. The overall prevalence of CKMS increased steadily from 1988 to 2018 in both sexes, with a larger temporal rise in prevalent stage 3 CKMS seen for men (from 18.9% to 22.4%) compared with women (from 13.9% to 15.2%).

Additional estimates of the prevalence of CKMS have been reported for South Korean and Chinese populations. Yim et al. [15] examined trends in the prevalence of CKMS in South Korea over the period of 2011–2021, using data from a nationwide study of South Korean adults aged ≥20 years. Stage 2 CKMS was the most prevalent (43.4%), followed by stages 1 (25.4%, 0 (21.1%), 3 (7.3%), and 4 (2.8%). Advanced stages showed significant increases (APC for stage 4: 3.2%; 95% CI, 1.5–5.2), while stage 0 declined (APC: −1.9%; 95% CI, −3.8 to 0.0). Hong et al. [16] analyzed the prevalence of CKMS stages in South Korea using data from the Korea National Health and Nutrition Examination Survey. Stage 0 was present in 25.2% of individuals, stage 1 in 19.3%, stage 2 in 51.6%, and stages 3–4 in 3.9%. The prevalence of stages 2 and 3–4 was higher in men than in women.

Zhang et al. [17] examined the prevalence of CKMS stages among middle-aged and older adults in China. The overall prevalence of CKM syndrome from stages 0 to 4 was 9.9%, 21.6%, 42.0%, 12.1%, and 14.4%, respectively. In men, the prevalence of CKM syndrome from stages 0 to 4 was 13.0%, 19.0%, 40.4%, 14.7%, and 12.8%, respectively. In women, the prevalence of CKM syndrome from stages 0 to 4 was 7.2%, 23.9%, 43.5%, 9.6%, and 15.8%, respectively. Finally, Huang et al. [18] examined the prevalence of CKMS in three cohort studies in China: the Pinggu study, the China Health and Retirement Longitudinal Study, and the 3B study. In the Pinggu study, 17.8%, 6.3%, and 5.3% of patients were at CKM stage 0 among people aged <45, 45–64, and ≥65 years, respectively. Overall, 1.5%, 12.3%, and 50.4% of patients had CKMS stages 3–4 across the three age categories. In the CHARLS study, a higher proportion of participants was at CKM stage 0, with 18.0% of individuals aged 45–64 years and 15.9% of those aged ≥65 years. Overall, 13.0% and 37.5% of individuals had CKMS stages 3–4 in these two age groups. In the 3B study, all patients were at least at stage 2, and stages 3 to 4 were observed in 8.9%, 28.9%, and 78.8% of patients aged <45, 45–64, and ≥65 years.

Our pooled sample size of the 2019, 2021, and 2023 BRFSS was 995,344, which was stratified into eight birth decades. Table 2 presents the prevalence rates of CKMS stages by birth decades. The prevalence of CKMS stage 4 was the highest among those born in the 1940s or before 1940—22.81% and 25.10%, respectively. The prevalence was the lowest among those born in the 1980s, 1990s, and after 1999—2.14%, 1.03%, and 0.55%, respectively (Supplemental Materials Figures S1 and S2).

The prevalence rates of CKMS stages by sex within birth decades are presented in Table 3. Among those born before the 1980s, males had a significantly higher rate of stage 4 CKMS compared to their female counterparts. The sex difference, however, was not statistically significant among those born in 2000 or later (Supplemental Materials Table S2).

Table 4 presents the rates by race and ethnicity within birth decades. Compared to their non-Hispanic white counterparts, non-Hispanic Blacks born during the 1940s, 1950s, 1960s, 1970s, 1980s, and 1990s had a significantly higher prevalence of CKMS stage 4. Prevalence among Hispanic adults born during 1950s, 1960s, 1970s, 1980s, and 1990s was also higher. With some exceptions, the prevalence rates among non-Hispanic Asians were generally lower than that of non-Hispanic Whites (Supplemental Material Table S3).

Prevalence rates by educational attainment and income are presented in Tables 5 and 6, respectively. In all birth decades, those who did not have a college degree generally have a higher prevalence of CKMS stage 4 (Supplemental Material Table S4). Similarly, among those born between the 1940s and 1990s, the prevalence of CKMS stage 4 in adults with lower household income was found to be higher (Supplemental Material Table S5). Lastly, Table 7 presents the prevalence rates by urban and rural residence. Except for those born in 2000 or later, adults residing in rural areas had a significantly higher prevalence of CKMS stage 4, compared to their counterparts living in urban areas (Supplemental Material Table S6). Distribution of age across birth decades and prevalence rates by selected age groups are presented in Supplemental Material Tables S7 and S8.

## 4. Discussion

To our knowledge, this study is among the first attempts to assess CKMS prevalence rates at the intersections of birth decade and sociodemographic and socioeconomic attributes including sex, race and ethnicity, education, income, and urban/rural residence, using large nationally representative data. The results of BRFSS data analysis indicate that CKMS is widespread in the general U.S. population, especially among older adults born during and before the 1960s (aged 50+ years). Within birth decades, it was found that males (compared to females), non-Hispanic Blacks and Hispanic individuals (compared to non-Hispanic Whites), adults with lower income and educational attainment, and adults living in rural areas (compared to adults living in urban areas) generally had higher prevalence rates of CKMS stage 4.

The results of the review indicate that the prevalence of CKMS is higher among Black adults as well as in South Korean and Chinese populations. A previous study found that 74.8% of Korean adults are at risk for all stages of CKMS, and rural individuals and those with lower education and income were more likely to have advanced stages [16]. Similarly, nearly 90% of Chinese adults aged 45–85 were classified as CKMS stages 1–4 [17]. Advanced CKMS was strongly associated with type 2 diabetes and obesity in different Chinese cohorts. China’s prevalence of advanced-stage CKMS rivals that of the U.S. population among adults aged ≥65 years [17]. However, South Korea’s advanced-stage prevalence of 3.9% remains well below the 14.6% observed in U.S. adults, which may be due to differences in dietary habits, lifestyle, and healthcare systems [16].

Notably, differences by sex were observed in CKMS prevalence and outcomes. The current study found that prevalence rates of stages 2–4 were higher in men than women, but both sexes showed a high disease burden. This trend was analyzed by Ji et al. [14], who reported a stage 3 prevalence of 22% in men compared to 15% in women, but women seemed to have equal or worse outcomes at any given level of CKMS. Given that CKMS reflects a complex interplay between cardiovascular, kidney, and metabolic conditions, it is important to recognize that women face many well-established risk factors for CKD and CVD [19–23]. In the BRFSS data, however, we observed a lower prevalence of advanced CKMS stages among women across birth decades. Further research is warranted to examine sex-specific risk factors for CKMS.

The findings underscore the importance of disaggregating CKMS data by demographic and socioeconomic attributes to better identify and respond to health disparities. Aggregated national estimates may obscure important subgroup differences, potentially overlooking populations in need of intervention and hindering targeted resource allocation. Future studies should explore the intersectionality of multiple sociodemographic and socioeconomic attributes in the context of CKMS, as certain groups may be subject to compounded risk due to the combined effects of longstanding disparities in access, utilization, and quality of care. Many individuals with CKMS do not present with classic risk factors such as longstanding diabetes or hypertension, and as a result, they may be underdiagnosed until later disease stages. Improved awareness and screening could facilitate earlier intervention and help slow disease progression. The presence of CKMS across demographic groups also highlights the need for expanded research into underlying causes, especially environmental exposures, psychosocial stressors, and barriers to preventive care, particularly among marginalized populations.

With respect to limitations, the BRFSS does not provide all information that constitutes clinical diagnosis of CKMS stages. In the absence of information (e.g., blood pressure and blood glucose levels, lipid profile, KDIGO classification), we utilized the best available proxies to construct measures for CKMS stages. A similar approach has been previously used in studies exploring CKMS outcomes using the BRFSS data [7]. Due to not having information about waist circumference, our estimates might include some adults with abdominal obesity in stages 0 and 1, and thereby potentially overestimated the prevalence of CKMS stages 0 and 1. Also, we did not have information about subclinical CVD, which is a key component of CKMS stage 3. As such, we had to combine CKMS stages 2 and 3, which is a major limitation of the study.

The BRFSS does not have information about levels of CKD risk (i.e., low risk, moderate to high risk, and very high risk). We only have information about whether the respondent had kidney disease (excluding kidney stones and bladder infection or incontinence). Risk level of CKD is a critical component in clinically determining CKMS stages. Absence of that information is another key limitation of the study.

In conclusion, a substantial proportion of the general U.S. population (in the BRFSS data) and selected Asian populations (in the literature review) meet the criteria for CKMS stages 1–4. Data suggested that prevalence of advanced CKMS stages is generally higher among older adults, males, and non-Hispanic Black individuals. Prevalence of CKMS stage 4 is also higher among adults from lower socioeconomic status conditions. These findings have implications for both policy and clinical practice. The importance of promptly defining and managing CKMS is emphasized by its widespread prevalence—affecting approximately 33–40% of U.S. adults—and by the fact that these inter-related conditions were among the leading causes of death in the United States in 2021 [2,3]. Risk stratification tools and clinical guidelines for CKD should incorporate race- and sex-specific considerations to better identify individuals at risk for CKMS. Given the progressive nature of CKMS and limited treatment options at advanced stages, there is an urgent need to focus on prevention through early identification of risk factors, lifestyle interventions, and improved access to primary care. To improve cardiovascular–kidney–metabolic health in the population, there is a need for an approach to CKMS staging that promotes prevention across the life course.

## Supplementary Material

Supplementary Material

**Supplementary Materials:** The following supporting information can be downloaded at https://www.mdpi.com/article/10.3390/cardiovascmed29010005/s1, Figure S1: Unadjusted predictions of CKMS stages by birth decade; Figure S2: Adjusted predictions of CKMS stages by birth decade; Table S1: Classification of CKMS stages in the BRFSS Data; Table S2. Relative risk ratios of CKMS stages for sex by birth decade; Table S3. Relative risk ratios of CKMS stages for race and ethnicity by birth decade; Table S4. Relative risk ratios of CKMS stages for education by birth decade; Table S5. Relative risk ratios of CKMS stages for income by birth decade; Table S6. Relative risk ratios of CKMS stages for urban/rural residence by birth decade; Table S7. Distribution of age by birth decade.; Table S8. Prevalence of CKMS stages by age group.

## Figures and Tables

**Table 1.** Studies on the prevalence of Cardiovascular–Kidney–Metabolic syndrome, 2023–2025.

Author	Study Population	Results	Limitations
Aggarwal et al. (2024) [3]	10,762 U.S. adults (51.8% women) who participated in NHANES 2011–2020	10.6% of U.S. adults met criteria for stage 0, 25.9% for stage 1, 49.0% for stage 2, 5.4% for stage 3, and 9.2% for stage 4 CKMS. The prevalence of each stage did not significantly change over the study period (*p* for trend > 0.05 for each stage). Adults 65 years or older were more likely to have advanced CKMS stages than those aged 45 to 64 years (55.3% vs. 10.7%, *p* < 0.001) or 20 to 44 years (53.3% vs. 2.1%, *p* < 0.001) (10). Compared with women, men were more likely to have advanced stages (16.9% vs. 12.4%, adjusted prevalence ratio [PR] = 1.36, 95% CI 1.24–1.49, *p* < 0.001), and Black adults were significantly more likely than white adults to have advanced-stage CKMS (18.9% vs. 13.8%, adjusted PR = 1.38, 95% CI 1.24–1.55, *p* < 0.001).	The study was limited by the use of self-reported information about CVD. Some CVD data used to define advanced-stage CKMS were unavailable.
Zhu et al. (2024) [11]	29,722 U.S. adults aged 30–79 years who participated in NHANES 1999–2018	The age-adjusted prevalence of CKMS stage 0 to 4 was 13.6%, 29.9%, 43.7%, 4.7%, and 8.1%, respectively. The highest proportion of stage 4 CKMS was among adults aged ≥65–79 years, males, and non-Hispanic Black individuals.	Some CVD data used to define advanced-stage CKMS were unavailable.
Minhas et al. (2024) [12]	U.S. Adults aged ≥20 years who participated in NHANES 2011 to 2018. Pregnant women were excluded.	Among those aged 20 to 44, 45 to 64, and ≥65 years, stage 0 CKM was present in 17.35%, 5.45%, and 1.80%, respectively, and stages 1–3 were present in 80.94%, 85.95%, and 72.03%, respectively. The highest proportion of stage 4 was among adults aged ≥65 years, males, and non-Hispanic white and non-Hispanic Black individuals.	The study was limited by the use of self-reported information about CVD. Some CVD data used to define advanced-stage CKMS were unavailable.
Kim et al. (2025) [13]	8474 U.S. adults aged ≥20 years who participated in NHANES 2011 to 2018. Pregnant women were excluded along with those with missing data to determine KMS stage components.	NHANES data from 2011 to 2018. The age-adjusted prevalence of stages 0–4 was 11.2%, 28.1%, 47.4%, 5.3%, and 8.1%, respectively. The highest proportion of stage 4 was among adults aged ≥60 years, males, and non-Hispanic Black individuals.	Some CVD data used to define advanced-stage CKMS were unavailable.
Ji et al. (2025) [14]	33,868 U.S. adults aged ≥20 years who participated in NHANES 1988 to 2018.	The overall prevalence of CKMS increased steadily from 1988 to 2018 in both sexes, with a larger temporal rise in prevalent stage 3 CKMS seen for men (from 18.9% to 22.4%) compared with women (from 13.9% to 15.2%).	Some CVD data used to define advanced-stage CKMS were unavailable.
Yim et al. (2025) [15]	61,106 South Korean adults aged ≥20 years who participated in Korea NHANES 2011 to 2021.	Over the period 2011–2021, stage 2 CKMS was the most prevalent (43.4%), followed by stages 1 (25.4%, 0 (21.1%), 3 (7.3%), and 4 (2.8%). Advanced stages showed significant increases (APC for stage 4: 3.2%; 95% CI, 1.5–5.2), while stage 0 declined (APC: −1.9%; 95% CI, −3.8 to 0.0).	The definition of CKMS stages was partially based upon the PREVENT equation, which should be validated in Asian populations.
Hong et al. (2025) [16]	54,994 Korean adults who participated in the Korea NHANES 2011 to 2021	Stage 0 was present in 25.2%, stage 1 in 19.3%, stage 2 in 51.6%, and stages 3–4 in 3.9% of patients. The prevalence of stages 2 and 3–4 was higher in men than in women.	Some CVD data used to define advanced-stage CKMS were unavailable. The CVD outcomes available in the Korea NHANES were limited to myocardial infarction, angina, and stroke.
Zhang et al. (2025) [17]	14,256 Chinese adults aged 45–85 years who participated in the China Health and Retirement Longitudinal Study.	The overall prevalence of CKM syndrome from stages 0 to 4 was 9.9%, 21.6%, 42.0%, 12.1%, and 14.4%, respectively. In men, the prevalence of CKM syndrome from stages 0 to 4 was 13.0%, 19.0%, 40.4%, 14.7%, and 12.8%, respectively. In women, the prevalence of CKM syndrome from stages 0 to 4 was 7.2%, 23.9%, 43.5%, 9.6%, and 15.8%, respectively.	The study was limited by the use of self-reported information about CVD. The definition of CKMS stages was partially based upon prediction equations, which should be validated in Asian populations.
Huang et al. (2025) [18]	4002 Chinese adults aged 25–75 years who participated in the Pinggu Study, 17,5000 Chinese adults >45 years who participated in the China Health and Retirement Longitudinal study; and 25,817 Chinese adults aged >18 years who participated in the 3B study.	In the Pinggu study, 17.8%, 6.3%, and 5.3% of patients were at CKM stage 0 among people aged <45, 45–64, and ≥65 years, respectively. Overall, 1.5%, 12.3%, and 50.4% had CKMS stages 3–4 across the 3 age categories. In the CHARLS study, a higher proportion of participants were at CKM stage 0, with 18.0% of individuals aged 45–64 years and 15.9% of those aged ≥65 years. Overall, 13.0% and 37.5% had CKMS stages 3–4 in these 2 age groups. In the 3B study, all patients were at least at stage 2, and stages 3 to 4 were observed in 8.9%, 28.9%, and 78.8% of patients aged <45, 45–64, and ≥65 years.	The study was limited by the use of self-reported information about CVD. The study populations were not representative of the overall Chinese population.

**Table 2.** Prevalence of CKMS stages by birth decade.

	CKMS Stages				Obs.

Birth Decade	Stage 0	Stage 1	Stage 2/3	Stage 4	

≤1939	10.92 (10.04, 11.80)	8.91 (8.11, 9.70)	55.08 (53.66, 56.49)	25.10 (23.94, 26.26)	29,294
1940s	7.94 (7.67, 8.21)	8.57 (8.29, 8.85)	60.68 (60.18, 61.18)	22.81 (22.38, 23.24)	200,708
1950s	9.29 (9.02, 9.57)	13.65 (13.30, 14.00)	62.21 (61.74, 62.68)	14.85 (14.52, 15.18)	230,173
1960s	12.05 (11.72, 12.37)	22.01 (21.59, 22.44)	56.16 (55.65, 56.66)	9.78 (9.48, 10.08)	185,831
1970s	16.17 (15.75, 16.59)	32.72 (32.18, 33.25)	46.07 (45.51, 46.64)	5.04 (4.76, 5.32)	136,828
1980s	22.64 (22.16, 23.12)	42.11 (41.55, 42.67)	33.11 (32.58, 33.64)	2.14 (1.98, 2.31)	115,188
1990s	33.46 (32.85, 34.08)	43.54 (42.89, 44.19)	21.97 (21.43, 22.51)	1.03 (0.89, 1.17)	77,196
≥2000	47.04 (45.77, 48.30)	34.19 (33.03, 35.35)	18.22 (17.16, 19.28)	0.55 (0.37, 0.74)	20,126

Note: Estimates were obtained using complex survey weights. The 95% confidence intervals are in parentheses.

**Table 3.** Prevalence of CKMS stages by birth decade and sex.

Birth Decade	CKMS Stages							

	Stage 0		Stage 1		Stage 2/3		Stage 4	

	Male	Female	Male	Female	Male	Female	Male	Female

≤1939	8.23 (7.03, 9.44)	12.75 (11.53, 13.98)	11.35 (9.85, 12.86)	7.23 (6.39, 8.07)	49.52 (47.26, 51.79)	58.88 (57.09, 60.67)	30.89 (28.93, 32.86)	21.14 (19.73, 22.54)
1940s	5.82 (5.46, 6.18)	9.68 (9.29, 10.06)	9.05 (8.63, 9.47)	8.18 (7.81, 8.54)	56.41 (55.65, 57.16)	64.17 (63.50, 64.84)	28.72 (28.05, 29.40)	17.98 (17.43, 18.53)
1950s	6.67 (6.33, 7.01)	11.85 (11.42, 12.28)	13.89 (13.42, 14.36)	13.42 (12.90, 13.93)	61.01 (60.34, 61.67)	63.38 (62.72, 64.04)	18.43 (17.91, 18.96)	11.35 (10.95, 11.75)
1960s	7.97 (7.61, 8.33)	16.06 (15.53, 16.60)	22.72 (22.10, 23.33)	21.32 (20.73, 21.90)	57.96 (57.26, 58.67)	54.38 (53.66, 55.09)	11.35 (10.91, 11.78)	8.24 (7.83, 8.66)
1970s	11.33 (10.81, 11.86)	21.09 (20.44, 21.73)	32.63 (31.88, 33.39)	32.80 (32.03, 33.57)	50.44 (49.63, 51.25)	41.64 (40.85, 42.42)	5.59 (5.14, 6.04)	4.48 (4.16, 4.80)
1980s	17.75 (17.14, 18.36)	27.56 (26.83, 28.29)	42.41 (41.61, 43.21)	41.81 (41.01, 42.60)	37.62 (36.86, 38.39)	28.56 (27.83, 29.29)	2.22 (1.98, 2.46)	2.07 (1.83, 2.30)
1990s	31.37 (30.55, 32.18)	35.69 (34.77, 36.60)	43.18 (42.30, 44.07)	43.92 (42.97, 44.87)	24.30 (23.53, 25.08)	19.49 (18.75, 20.23)	1.15 (0.93, 1.37)	0.90 (0.75, 1.06)
≥2000	47.32 (45.68, 48.96)	46.73 (44.79, 48.68)	34.69 (33.16, 36.23)	33.65 (31.89, 35.41)	17.42 (16.20, 18.64)	19.09 (17.32, 20.85)	0.57 (0.34, 0.80)	0.53 (0.24, 0.83)

Note: Estimates were obtained using complex survey weights. The 95% confidence intervals are in parentheses.

**Table 4.** Prevalence of CKMS stages by birth decade and race and ethnicity.

Birth Decade	White	Black	Hispanic	Asian	Other

**Stage 0**					
≤1939	11.41 (10.51, 12.31)	7.11 (3.80, 10.43)	6.57 (3.88, 9.26)	17.14 (6.29, 27.99)	10.01 (4.93, 15.10)
1940s	8.60 (8.31, 8.90)	3.56 (2.88, 4.24)	4.92 (3.67, 6.17)	9.16 (6.86, 11.46)	8.61 (7.20, 10.03)
1950s	10.30 (10.01, 10.59)	4.71 (4.09, 5.33)	5.75 (4.70, 6.79)	12.29 (9.39, 15.19)	8.87 (7.62, 10.11)
1960s	13.36 (13.02, 13.70)	6.56 (5.63, 7.48)	8.21 (7.09, 9.32)	19.11 (16.41, 21.81)	11.30 (10.08, 12.52)
1970s	17.92 (17.47, 18.38)	9.99 (9.06, 10.93)	11.42 (10.23, 12.62)	26.92 (24.25, 29.59)	14.91 (13.08, 16.73)
1980s	24.76 (24.20, 25.32)	15.60 (14.35, 16.86)	16.38 (15.24, 17.52)	36.87 (34.38, 39.36)	20.98 (18.95, 23.00)
1990s	35.37 (34.62, 36.12)	28.45 (26.57, 30.32)	27.74 (26.26, 29.22)	46.03 (43.56, 48.49)	30.79 (28.45, 33.13)
≥2000	48.59 (47.02, 50.17)	45.89 (42.17, 49.61)	39.56 (36.82, 42.29)	62.22 (57.24, 67.21)	42.87 (38.27, 47.47)
**Stage 1**					
≤1939	9.44 (8.61, 10.26)	5.22 (3.23, 7.20)	9.73 (3.08, 16.38)	3.61 (1.41, 5.81)	8.76 (5.08, 12.44)
1940s	8.74 (8.46, 9.02)	5.74 (4.92, 6.57)	10.61 (8.80, 12.41)	7.09 (4.61, 9.57)	9.21 (7.73, 10.70)
1950s	13.98 (13.65, 14.30)	10.44 (9.40, 11.47)	15.81 (13.68, 17.94)	11.16 (8.86, 13.45)	14.17 (12.43, 15.91)
1960s	22.30 (21.86, 22.73)	19.25 (17.92, 20.59)	24.82 (23.16, 26.47)	17.59 (14.82, 20.37)	22.00 (20.40, 23.60)
1970s	32.82 (32.24, 33.39)	31.96 (30.42, 33.51)	36.13 (34.42, 37.84)	24.78 (22.25, 27.32)	32.94 (30.70, 35.17)
1980s	41.08 (40.45, 41.72)	45.03 (43.37, 46.69)	48.31 (46.76, 49.87)	29.38 (27.09, 31.66)	40.08 (37.79, 42.37)
1990s	42.12 (41.36, 42.89)	49.20 (47.21, 51.18)	47.51 (45.86, 49.17)	32.32 (29.95, 34.69)	45.24 (42.40, 48.08)
≥2000	33.60 (32.11, 35.09)	37.82 (34.36, 41.28)	39.38 (36.68, 42.09)	19.47 (16.00, 22.93)	34.47 (30.30, 38.64)
**Stage 2/3**					
≤1939	53.09 (51.69, 54.49)	65.85 (60.77, 70.93)	61.16 (52.16, 70.16)	60.02 (47.58, 72.46)	60.42 (52.00, 68.84)
1940s	59.30 (58.79, 59.81)	68.51 (66.71, 70.31)	67.19 (64.41, 69.98)	64.95 (60.48, 69.42)	56.13 (53.53, 58.73)
1950s	61.23 (60.78, 61.69)	67.41 (65.96, 68.85)	64.35 (61.89, 66.81)	64.82 (60.91, 68.73)	57.82 (55.65, 59.99)
1960s	55.06 (54.55, 55.57)	61.59 (60.00, 63.18)	57.88 (55.96, 59.81)	55.65 (52.14, 59.17)	52.81 (50.82, 54.80)
1970s	44.47 (43.87, 45.08)	51.05 (49.41, 52.68)	48.15 (46.41, 49.88)	45.69 (42.80, 48.59)	43.76 (41.33, 46.19)
1980s	32.30 (31.71, 32.90)	36.48 (34.89, 38.07)	32.90 (31.44, 34.36)	32.04 (29.71, 34.37)	35.99 (33.78, 38.21)
1990s	21.71 (21.08, 22.34)	20.97 (19.47, 22.46)	23.52 (22.07, 24.98)	21.19 (19.12, 23.26)	21.33 (19.42, 23.23)
≥2000	17.26 (16.06, 18.45)	15.74 (13.28, 18.20)	20.38 (17.81, 22.96)	18.09 (13.51, 22.67)	22.04 (17.58, 26.50)
**Stage 4**					
≤1939	26.06 (24.84, 27.27)	21.82 (17.95, 25.69)	22.54 (15.27, 29.80)	19.23 (10.58, 27.88)	20.81 (15.62, 26.00)
1940s	23.36 (22.91, 23.80)	22.19 (20.55, 23.83)	17.28 (15.15, 19.41)	18.80 (15.42, 22.18)	26.04 (23.66, 28.43)
1950s	14.49 (14.16, 14.82)	17.45 (16.30, 18.59)	14.09 (12.44, 15.74)	11.73 (9.27, 14.19)	19.15 (17.54, 20.75)
1960s	9.29 (8.99, 9.59)	12.60 (11.51, 13.69)	9.09 (8.04, 10.14)	7.64 (5.58, 9.70)	13.89 (12.64, 15.13)
1970s	4.79 (4.50, 5.07)	6.99 (6.19, 7.79)	4.30 (3.62, 4.97)	2.60 (1.75, 3.45)	8.40 (5.70, 11.09)
1980s	1.86 (1.69, 2.02)	2.89 (2.35, 3.42)	2.41 (1.88, 2.93)	1.71 (1.06, 2.37)	2.95 (2.40, 3.50)
1990s	0.80 (0.68, 0.91)	1.39 (0.82, 1.96)	1.22 (0.90, 1.54)	0.46 (0.20, 0.73)	2.64 (1.39, 3.89)
≥2000	0.55 (0.31, 0.79)	0.55 (−0.03, 1.13)	0.68 (0.19, 1.17)	0.22 (−0.00, 0.44)	0.62 (0.21, 1.02)

Note: Estimates were obtained using complex survey weights. The 95% confidence intervals are in parentheses.

**Table 5.** Prevalence of CKMS stages by birth decade and education.

Birth Decade	High School or Less	Some College	College Degree

**Stage 0**			
≤1939	9.43 (8.17, 10.69)	11.31 (9.57, 13.05)	13.69 (12.00, 15.38)
1940s	6.64 (6.21, 7.08)	7.83 (7.31, 8.34)	9.84 (9.41, 10.27)
1950s	7.03 (6.62, 7.44)	8.91 (8.41, 9.41)	12.36 (11.84, 12.89)
1960s	9.10 (8.56, 9.64)	10.44 (9.81, 11.08)	16.73 (16.19, 17.27)
1970s	11.40 (10.67, 12.13)	13.37 (12.64, 14.11)	22.36 (21.67, 23.05)
1980s	17.16 (16.35, 17.97)	20.08 (19.13, 21.02)	29.03 (28.30, 29.77)
1990s	28.94 (27.84, 30.05)	33.19 (32.03, 34.34)	38.49 (37.60, 39.38)
≥2000	47.42 (45.73, 49.12)	46.91 (44.77, 49.05)	44.51 (40.75, 48.26)
**Stage 1**			
≤1939	9.11 (7.87, 10.36)	8.74 (7.19, 10.30)	8.55 (7.41, 9.69)
1940s	8.17 (7.71, 8.63)	8.47 (7.95, 8.98)	9.19 (8.74, 9.65)
1950s	13.33 (12.64, 14.01)	13.37 (12.78, 13.97)	14.31 (13.82, 14.80)
1960s	20.56 (19.83, 21.30)	22.99 (22.16, 23.83)	22.75 (22.11, 23.38)
1970s	32.00 (30.99, 33.01)	33.92 (32.86, 34.98)	32.39 (31.65, 33.14)
1980s	44.72 (43.62, 45.82)	43.65 (42.54, 44.75)	38.85 (38.08, 39.62)
1990s	46.58 (45.35, 47.80)	45.00 (43.81, 46.20)	38.84 (37.95, 39.72)
≥2000	34.08 (32.53, 35.63)	34.16 (32.17, 36.14)	35.39 (31.76, 39.03)
**Stage 2/3**			
≤1939	54.93 (52.77, 57.08)	55.68 (52.93, 58.43)	54.72 (52.45, 56.99)
1940s	60.63 (59.76, 61.51)	60.29 (59.36, 61.22)	61.22 (60.48, 61.96)
1950s	61.43 (60.57, 62.29)	62.29 (61.46, 63.11)	63.05 (62.33, 63.77)
1960s	56.96 (56.09, 57.83)	56.47 (55.50, 57.43)	55.06 (54.27, 55.84)
1970s	49.09 (48.01, 50.16)	47.34 (46.25, 48.43)	42.74 (41.96, 43.53)
1980s	34.79 (33.74, 35.85)	34.16 (33.13, 35.18)	30.92 (30.19, 31.65)
1990s	22.71 (21.66, 23.76)	21.02 (20.09, 21.95)	22.17 (21.40, 22.94)
≥2000	17.83 (16.41, 19.25)	18.51 (16.71, 20.31)	19.91 (16.64, 23.17)
**Stage 4**			
≤1939	26.52 (24.72, 28.33)	24.26 (22.10, 26.43)	23.04 (21.18, 24.90)
1940s	24.55 (23.80, 25.31)	23.42 (22.61, 24.22)	19.75 (19.15, 20.34)
1950s	18.21 (17.57, 18.85)	15.43 (14.84, 16.03)	10.28 (9.86, 10.70)
1960s	13.37 (12.81, 13.94)	10.10 (9.55, 10.65)	5.46 (5.08, 5.85)
1970s	7.51 (6.95, 8.07)	5.36 (4.91, 5.82)	2.51 (2.29, 2.73)
1980s	3.33 (2.91, 3.74)	2.12 (1.84, 2.39)	1.20 (1.03, 1.37)
1990s	1.77 (1.42, 2.12)	0.79 (0.64, 0.94)	0.50 (0.38, 0.62)
≥2000	0.67 (0.39, 0.95)	0.42 (0.19, 0.65)	0.19 (−0.01, 0.40)

Note: Estimates were obtained using complex survey weights. The 95% confidence intervals are in parentheses.

**Table 6.** Prevalence of CKMS stages by birth decade and income.

Birth Decade	Low	Middle	High

**Stage 0**			
≤1939	8.72 (7.20, 10.25)	10.40 (8.72, 12.07)	9.90 (8.12, 11.69)
1940s	6.57 (6.02, 7.13)	7.40 (6.95, 7.85)	8.54 (8.01, 9.07)
1950s	7.06 (6.56, 7.56)	8.74 (8.20, 9.29)	10.55 (10.04, 11.06)
1960s	8.85 (8.09, 9.60)	12.20 (11.61, 12.79)	13.26 (12.71, 13.82)
1970s	11.91 (10.94, 12.87)	18.05 (17.38, 18.71)	16.11 (15.36, 16.86)
1980s	18.25 (17.35, 19.15)	23.71 (22.92, 24.50)	25.57 (24.57, 26.57)
1990s	31.77 (30.60, 32.94)	31.89 (30.80, 32.98)	36.41 (35.20, 37.62)
≥2000	43.00 (40.73, 45.27)	49.49 (47.06, 51.91)	50.11 (46.47, 53.75)
**Stage 1**			
≤1939	8.01 (6.46, 9.56)	10.15 (8.48, 11.82)	8.99 (7.27, 10.71)
1940s	7.61 (7.01, 8.21)	8.71 (8.23, 9.18)	9.33 (8.73, 9.92)
1950s	11.63 (10.87, 12.39)	13.80 (13.18, 14.41)	15.70 (15.01, 16.39)
1960s	17.57 (16.69, 18.44)	23.94 (23.12, 24.76)	23.77 (23.04, 24.51)
1970s	30.41 (29.27, 31.55)	34.07 (33.23, 34.91)	33.45 (32.40, 34.50)
1980s	43.47 (42.35, 44.58)	43.15 (42.24, 44.06)	39.95 (38.82, 41.09)
1990s	45.03 (43.81, 46.25)	44.99 (43.80, 46.18)	39.68 (38.46, 40.91)
≥2000	38.11 (35.90, 40.31)	31.94 (29.78, 34.10)	32.60 (29.29, 35.91)
**Stage 2/3**			
≤1939	55.76 (53.12, 58.41)	54.60 (52.02, 57.18)	56.24 (52.69, 59.79)
1940s	58.97 (57.85, 60.10)	61.32 (60.44, 62.20)	61.41 (60.44, 62.37)
1950s	59.65 (58.62, 60.68)	63.28 (62.42, 64.14)	63.64 (62.81, 64.47)
1960s	56.37 (55.26, 57.48)	56.02 (55.07, 56.97)	57.03 (56.19, 57.87)
1970s	48.06 (46.83, 49.29)	44.79 (43.91, 45.67)	47.15 (46.05, 48.25)
1980s	34.48 (33.42, 35.54)	31.71 (30.87, 32.56)	33.42 (32.36, 34.49)
1990s	21.63 (20.62, 22.65)	22.36 (21.31, 23.40)	23.27 (22.21, 24.32)
≥2000	18.15 (16.18, 20.11)	18.20 (16.14, 20.26)	16.97 (14.15, 19.79)
**Stage 4**			
≤1939	27.50 (25.29, 29.72)	24.86 (22.68, 27.03)	24.87 (21.88, 27.86)
1940s	26.85 (25.88, 27.83)	22.57 (21.83, 23.31)	20.73 (19.92, 21.54)
1950s	21.67 (20.84, 22.49)	14.18 (13.59, 14.76)	10.11 (9.63, 10.60)
1960s	17.22 (16.47, 17.96)	7.85 (7.30, 8.39)	5.93 (5.52, 6.35)
1970s	9.62 (8.91, 10.33)	3.10 (2.80, 3.39)	3.29 (2.88, 3.71)
1980s	3.81 (3.37, 4.24)	1.43 (1.20, 1.66)	1.05 (0.86, 1.25)
1990s	1.56 (1.29, 1.83)	0.76 (0.51, 1.01)	0.64 (0.35, 0.93)
≥2000	0.75 (0.40, 1.10)	0.38 (0.12, 0.63)	0.33 (0.00, 0.65)

Note: Estimates were obtained using complex survey weights. The 95% confidence intervals are in parentheses.

**Table 7.** Prevalence of CKMS stages by birth decade and urban/rural residence.

Birth Decade	CKMS Stages							

	Stage 0		Stage 1		Stage 2/3		Stage 4	

	Urban	Rural	Urban	Rural	Urban	Rural	Urban	Rural

≤1939	11.07 (10.04, 12.09)	10.17 (8.87, 11.48)	9.01 (8.08, 9.94)	8.38 (7.25, 9.52)	55.35 (53.72, 56.98)	53.72 (51.46, 55.99)	24.57 (23.25, 25.90)	27.72 (25.60, 29.84)
1940s	8.11 (7.80, 8.42)	7.16 (6.71, 7.61)	8.56 (8.24, 8.88)	8.62 (8.13, 9.11)	61.09 (60.51, 61.66)	58.76 (57.85, 59.68)	22.25 (21.76, 22.73)	25.46 (24.59, 26.32)
1950s	9.43 (9.11, 9.75)	8.65 (8.22, 9.09)	13.72 (13.31, 14.13)	13.32 (12.79, 13.85)	62.50 (61.95, 63.05)	60.83 (60.07, 61.59)	14.35 (13.97, 14.73)	17.20 (16.61, 17.79)
1960s	12.39 (12.01, 12.76)	10.17 (9.64, 10.70)	22.29 (21.80, 22.77)	20.50 (19.76, 21.23)	56.12 (55.54, 56.69)	56.38 (55.49, 57.27)	9.21 (8.87, 9.55)	12.96 (12.36, 13.55)
1970s	16.68 (16.21, 17.15)	12.89 (12.20, 13.57)	32.87 (32.27, 33.48)	31.71 (30.75, 32.67)	45.74 (45.11, 46.37)	48.22 (47.16, 49.28)	4.71 (4.40, 5.01)	7.18 (6.66, 7.71)
1980s	23.18 (22.65, 23.71)	18.80 (17.90, 19.70)	42.02 (41.40, 42.64)	42.71 (41.55, 43.88)	32.75 (32.16, 33.33)	35.70 (34.56, 36.84)	2.05 (1.87, 2.24)	2.79 (2.42, 3.16)
1990s	34.23 (33.56, 34.90)	27.48 (26.19, 28.77)	42.94 (42.24, 43.65)	48.18 (46.70, 49.65)	21.82 (21.24, 22.41)	23.09 (21.79, 24.39)	1.00 (0.85, 1.15)	1.26 (1.00, 1.52)
≥2000	47.55 (46.17, 48.93)	43.32 (40.36, 46.28)	33.64 (32.38, 34.89)	38.22 (35.27, 41.17)	18.29 (17.12, 19.45)	17.74 (15.50, 19.98)	0.53 (0.33, 0.73)	0.72 (0.32, 1.13)

Note: Estimates were obtained using complex survey weights. The 95% confidence intervals are in parentheses.

## Data Availability

The original contributions presented in this study are included in the article/Supplementary Materials. Further inquiries can be directed to the corresponding author. BRFSS data are publicly available (https://www.cdc.gov/brfss/ (accessed on 16 November 2025)).
