# Supplementary material for: The Prevalence of Cardiovascular–Kidney–Metabolic Syndrome: A Review of Published Estimates and New Findings from BRFSS Surveys
Source: Cardiovasc Med (Basel). Author manuscript; Available in PMC 2026 Aug 4. (PMC13435001; doi:10.3390/cardiovascmed29010005)
Supplement: Supplementary Material [file NIHMS2192640-supplement-Supplementary_Material.zip › cardiovascmed-4021818-supplementary.pdf]

Supplemental Materials

Figure S1. Unadjusted predictions of CKMS stages by birth decade.

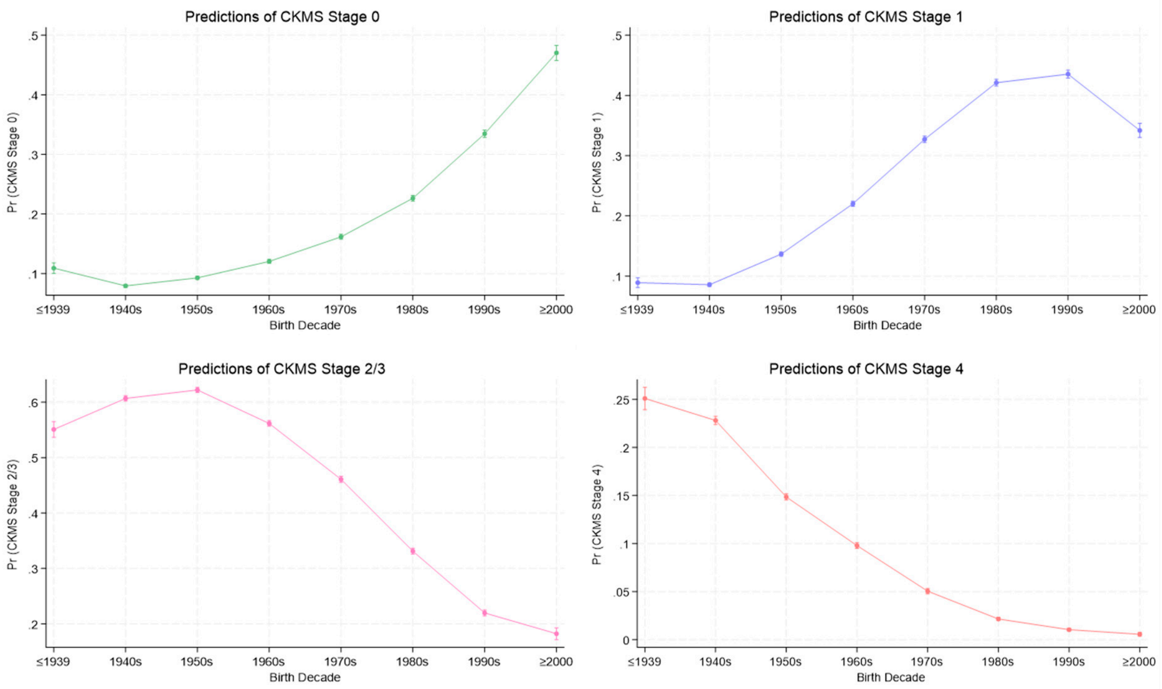

Note: Estimates were from multinomial logistic regression using complex survey weights. CKMS stage 0 was the base category. Vertical lines across the markers represent 95% confidence intervals.

Figure S2. Adjusted predictions of CKMS stages by birth decade.

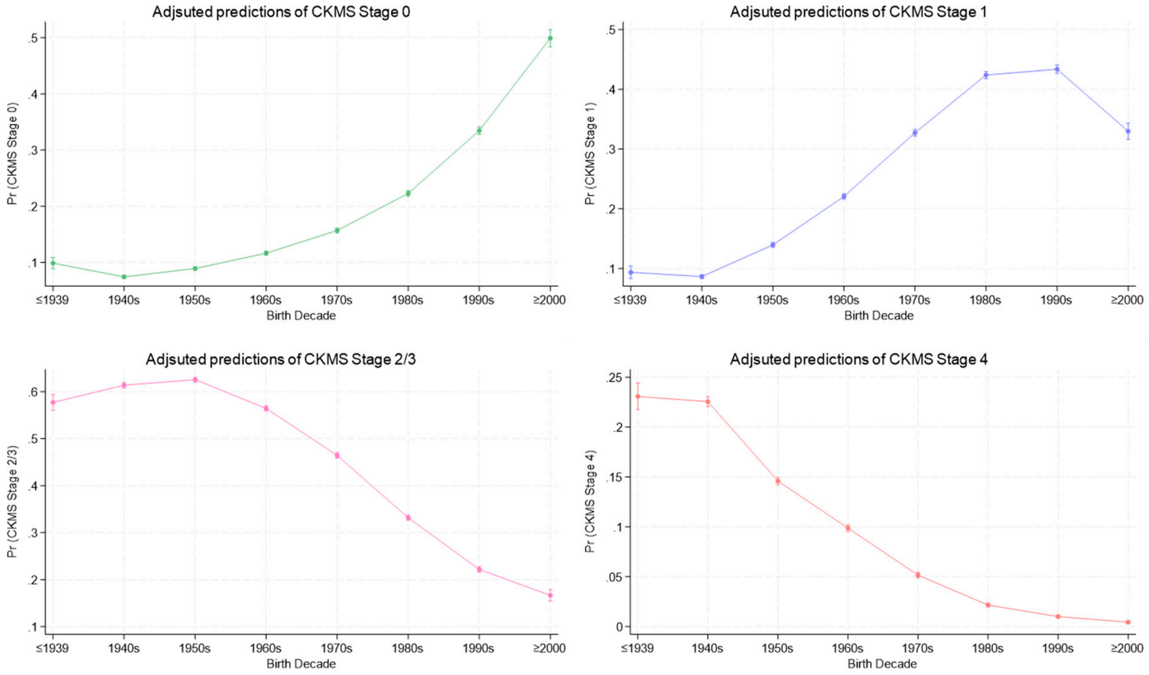

Note: Estimates were from multinomial logistic regression using complex survey weights. CKMS stage 0 was the base category. Adjustments were made for sex, race and ethnicity, educational attainment, income, and urban/rural residence. Vertical lines across the markers represent 95% confidence intervals.

Table S1. Classification of CKMS stages in the BRFSS Data

	Criteria stated by Li et al., 2024	BRFSS
**Stage 0**: No CKM risk factors<br><br>Individuals with normal BMI and waist circumference, normoglycemia, normotension, a normal lipid profile, and no evidence of CKD or subclinical or clinical CVD	*All of the following*:<br><br>1) BMI <25 kg/m^2 (or <23 kg/m^2 if Asian ancestry)<br>2) Waist circumference <88/102 cm in female/male (or if Asian ancestry <80/90 cm in female/male)<br>3) Fasting blood glucose < 100 mg/dL and HbA1c < 5.7% and without self-reported diagnosis of diabetes, use of insulin, or oral hypoglycemic agents<br>4) SBP <130 mm Hg and DBP <80 mm Hg without self-reported diagnosis of hypertension or use of antihypertensive medications<br>5) HDL cholesterol <50/40 mg/dL in female/male and triglycerides < 150 mg/dL<br>6) Low-risk CKD in KDIGO classification according to eGFR and UACR: UACR < 30 mg/g and eGFR ≥ 60 ml/min/1.73m2.<br>7) Predicted 10-year CVD risk < 20%<br>8) No clinical CVD	**Stage 0**:<br><br>• BMI <25 kg/m^2<br>• No diabetes<br>• No pre-diabetes<br>• No hypertension<br>• No pre-hypertension<br>• No high cholesterol<br>• No CKD<br>• No CVD
**Stage 1**: Excess or dysfunctional adiposity<br><br>Individuals with overweight/obesity, abdominal obesity, or dysfunctional adipose tissue, without the presence of other metabolic risk factors or CKD	*Any of the 3*:<br><br>1) Overweight/obesity<br>2) Abdominal obesity<br>3) Prediabetes<br><br>*All of the following*:<br><br>1) SBP <130 mm Hg and DBP <80 mm Hg without self-reported diagnosis of hypertension or use of antihypertensive medications<br>2) HDL cholesterol <50/40 mg/dL in female/male and triglycerides < 150 mg/dL<br>3) Low-risk CKD in KDIGO classification according to eGFR and UACR: UACR < 30 mg/g and eGFR ≥ 60 ml/min/1.73m2.<br>4) Predicted 10-year CVD risk < 20%<br>5) No clinical CVD	**Stage 1**:<br>• BMI >25 kg/m^2, or<br>• Pre-diabetes<br><br><u>And</u>:<br>• No diabetes<br>• No hypertension<br>• No high cholesterol<br>• No CKD<br>• No CVD
**Stage 2**: Metabolic risk factors and CKD<br><br>Individuals with metabolic risk factors (hypertriglyceridemia, hypertension, MetS, diabetes), or CKD	*Any of the 5*:<br><br>1) Hypertriglyceridemia<br>2) Hypertension<br>3) Diabetes<br>4) MetS<br>5) Moderate-to-high-risk CKD in KDIGO classification	**Stage 2 and 3**:<br>• High cholesterol, or<br>• Hypertension, or<br>• Diabetes, or<br>• MetS (any 3 of the: overweight/obesity, diabetes,

	All of the following:<br><br>1) No very high-risk CKD in KDIGO classification<br>2) Predicted 10-year CVD risk < 20%<br>3) No clinical CVD	hypertension, high cholesterol), or<br>• Overweight/obesity, or<br>• Pre-diabetes, or<br>• CKD<br><br>And:<br>• No CVD
**Stage 3**: Subclinical CVD in CKM<br><br>Subclinical CVD among individuals with excess/dysfunctional adiposity, other metabolic risk factors, or CKD	*Any of the 2*:<br><br>1) Very high-risk CKD in KDIGO classification<br>2) Predicted 10-year CVD risk ≥ 20%<br><br>*Any of the 8*:<br><br>1) Overweight/obesity<br>2) Abdominal obesity<br>3) Prediabetes<br>4) Hypertriglyceridemia<br>5) Hypertension<br>6) diabetes<br>7) MetS<br>8) Moderate-to-high-risk CKD in KDIGO classification<br><br>*All of the following*:<br><br>No clinical CVD	
**Stage 4**: Clinical CVD in CKM<br><br>Clinical CVD among individuals with excess/dysfunctional adiposity, other metabolic risk factors, or CKD	*Any of the 9*:<br><br>1) Overweight/obesity<br>2) Abdominal obesity<br>3) Prediabetes<br>4) Hypertriglyceridemia<br>5) Hypertension<br>6) diabetes<br>7) MetS<br>8) Moderate-to-high-risk CKD in KDIGO classification<br>9) Very high-risk CKD in KDIGO classification<br><br>*All of the following*:<br><br>Clinical CVD	**Stage 4**:<br>• Overweight/obesity, or<br>• Diabetes, or<br>• Pre-diabetes, or<br>• Hypertension, or<br>• High cholesterol, or<br>• MetS, or<br>• CKD<br><br>And:<br>CVD

Table S2. Relative risk ratios of CKMS stages for sex by birth decade.

	**Base outcome**: Stage 0	**Outcome 1**: Stage 1	**Outcome 2**: Stage 2/3	**Outcome 3**: Stage 4
**≤1939**				
Male		Ref.	Ref.	Ref.
Female		0.41***	0.77*	0.44***
		(0.32, 0.53)	(0.63, 0.94)	(0.36, 0.55)
**1940s**				
Male		Ref.	Ref.	Ref.
Female		0.54***	0.68***	0.38***
		(0.49, 0.60)	(0.63, 0.74)	(0.34, 0.41)
**1950s**				
Male		Ref.	Ref.	Ref.
Female		0.54***	0.58***	0.35***
		(0.50, 0.59)	(0.54, 0.63)	(0.32, 0.38)
**1960s**				
Male		Ref.	Ref.	Ref.
Female		0.47***	0.47***	0.36***
		(0.43, 0.50)	(0.44, 0.50)	(0.33, 0.39)
**1970s**				
Male		Ref.	Ref.	Ref.
Female		0.54***	0.44***	0.43***
		(0.50, 0.58)	(0.41, 0.48)	(0.38, 0.49)
**1980s**				
Male		Ref.	Ref.	Ref.
Female		0.63***	0.49***	0.60***
		(0.60, 0.67)	(0.46, 0.52)	(0.51, 0.71)
**1990s**				
Male		Ref.	Ref.	Ref.
Female		0.89***	0.70***	0.69**
		(0.84, 0.95)	(0.66, 0.76)	(0.53, 0.90)
**≥2000**				
Male		Ref.	Ref.	Ref.
Female		0.98	1.11	0.95
		(0.88, 1.10)	(0.95, 1.29)	(0.48, 1.88)

Note: Estimates were obtained using complex survey weights. 95% confidence intervals are in parenthesis. *** $p < 0.001$, ** $p < 0.01$, * $p < 0.05$.

Table S3. Relative risk ratios of CKMS stages for race and ethnicity by birth decade.

	**Base outcome**: Stage 0	**Outcome 1**: Stage 1	**Outcome 2**: Stage 2/3	**Outcome 3**: Stage 4
**≤1939**				
White		Ref.	Ref.	Ref.
Black		0.89	1.99**	1.34
		(0.47, 1.67)	(1.18, 3.35)	(0.79, 2.28)
Hispanic		1.79	2.00**	1.50
		(0.78, 4.14)	(1.25, 3.21)	(0.88, 2.56)
Asian		0.25**	0.75	0.49
		(0.10, 0.64)	(0.34, 1.67)	(0.21, 1.16)
Other		1.06	1.30	0.91
		(0.54, 2.09)	(0.71, 2.38)	(0.51, 1.61)
**1940s**				
White		Ref.	Ref.	Ref.
Black		1.59***	2.79***	2.30***
		(1.24, 2.04)	(2.28, 3.43)	(1.85, 2.85)
Hispanic		2.12***	1.98***	1.29
		(1.54, 2.92)	(1.51, 2.61)	(0.97, 1.74)
Asian		0.76	1.03	0.76
		(0.49, 1.19)	(0.77, 1.38)	(0.55, 1.04)
Other		1.05	0.95	1.11
		(0.82, 1.35)	(0.78, 1.14)	(0.91, 1.37)
**1950s**				
White		Ref.	Ref.	Ref.
Black		1.63***	2.41***	2.63***
		(1.37, 1.95)	(2.09, 2.78)	(2.25, 3.08)
Hispanic		2.03***	1.88***	1.74***
		(1.59, 2.58)	(1.54, 2.30)	(1.39, 2.19)
Asian		0.67*	0.89	0.68*
		(0.48, 0.93)	(0.67, 1.17)	(0.48, 0.95)
Other		1.18	1.10	1.53***
		(0.96, 1.44)	(0.93, 1.29)	(1.29, 1.83)
**1960s**				
White		Ref.	Ref.	Ref.
Black		1.76***	2.28***	2.77***
		(1.48, 2.09)	(1.95, 2.66)	(2.31, 3.31)
Hispanic		1.81***	1.71***	1.59***
		(1.54, 2.14)	(1.47, 2.00)	(1.32, 1.93)
Asian		0.55***	0.71***	0.58***
		(0.44, 0.70)	(0.59, 0.85)	(0.42, 0.80)
Other		1.17*	1.13	1.77***
		(1.01, 1.35)	(0.99, 1.29)	(1.52, 2.06)
**1970s**				
White		Ref.	Ref.	Ref.
Black		1.75***	2.06***	2.62***
		(1.55, 1.97)	(1.84, 2.30)	(2.22, 3.10)
Hispanic		1.73***	1.70***	1.41**
		(1.51, 1.97)	(1.50, 1.93)	(1.15, 1.73)
Asian		0.50***	0.68***	0.36***
		(0.42, 0.60)	(0.59, 0.79)	(0.25, 0.52)
Other		1.21*	1.18*	2.11***
		(1.03, 1.41)	(1.02, 1.38)	(1.45, 3.08)
**1980s**				

White	Ref.	Ref.	Ref.
Black	1.74***	1.79***	2.47***
	(1.56, 1.93)	(1.61, 2.00)	(1.96, 3.10)
Hispanic	1.78***	1.54***	1.96***
	(1.62, 1.95)	(1.39, 1.70)	(1.52, 2.52)
Asian	0.48***	0.67***	0.62*
	(0.42, 0.55)	(0.59, 0.76)	(0.41, 0.93)
Other	1.15*	1.32***	1.88***
	(1.00, 1.32)	(1.14, 1.51)	(1.48, 2.38)
**1990s**			
White	Ref.	Ref.	Ref.
Black	1.45***	1.20**	2.17***
	(1.31, 1.61)	(1.07, 1.35)	(1.39, 3.40)
Hispanic	1.44***	1.38***	1.95***
	(1.32, 1.57)	(1.24, 1.53)	(1.43, 2.67)
Asian	0.59***	0.75***	0.45**
	(0.52, 0.66)	(0.65, 0.86)	(0.25, 0.81)
Other	1.23**	1.13	3.81***
	(1.08, 1.41)	(0.99, 1.29)	(2.28, 6.37)
**≥2000**			
White	Ref.	Ref.	Ref.
Black	1.19	0.97	1.07
	(1.00, 1.42)	(0.77, 1.21)	(0.34, 3.36)
Hispanic	1.44***	1.45***	1.52
	(1.25, 1.66)	(1.20, 1.76)	(0.65, 3.56)
Asian	0.45***	0.82	0.31*
	(0.36, 0.57)	(0.59, 1.14)	(0.10, 0.93)
Other	1.16	1.45*	1.28
	(0.94, 1.43)	(1.08, 1.94)	(0.58, 2.83)

Note: Estimates were obtained using complex survey weights. 95% confidence intervals are in parenthesis. *** $p < 0.001$, ** $p < 0.01$, * $p < 0.05$.

Table S4. Relative risk ratios of CKMS stages for education by birth decade.

	**Base outcome**: Stage 0	**Outcome 1**: Stage 1	**Outcome 2**: Stage 2/3	**Outcome 3**: Stage 4
**≤1939**				
High school or less		Ref.	Ref.	Ref.
Some college		0.80	0.85	0.76*
		(0.58, 1.10)	(0.67, 1.07)	(0.60, 0.98)
College degree		0.65**	0.69***	0.60***
		(0.49, 0.85)	(0.55, 0.85)	(0.48, 0.75)
**1940s**				
High school or less		Ref.	Ref.	Ref.
Some college		0.88	0.84**	0.81***
		(0.77, 1.00)	(0.76, 0.93)	(0.73, 0.90)
College degree		0.76***	0.68***	0.54***
		(0.68, 0.85)	(0.62, 0.74)	(0.49, 0.60)
**1950s**				
High school or less		Ref.	Ref.	Ref.
Some college		0.79***	0.80***	0.67***
		(0.71, 0.89)	(0.73, 0.87)	(0.60, 0.74)
College degree		0.61***	0.58***	0.32***
		(0.55, 0.67)	(0.54, 0.63)	(0.29, 0.35)
**1960s**				
High school or less		Ref.	Ref.	Ref.
Some college		0.97	0.86**	0.66***
		(0.88, 1.08)	(0.78, 0.95)	(0.59, 0.74)
College degree		0.60***	0.53***	0.22***
		(0.55, 0.66)	(0.49, 0.57)	(0.20, 0.25)
**1970s**				
High school or less		Ref.	Ref.	Ref.
Some college		0.90	0.82***	0.61***
		(0.81, 1.00)	(0.74, 0.91)	(0.53, 0.71)
College degree		0.52***	0.44***	0.17***
		(0.47, 0.57)	(0.41, 0.48)	(0.15, 0.20)
**1980s**				
High school or less		Ref.	Ref.	Ref.
Some college		0.83***	0.84***	0.54***
		(0.76, 0.91)	(0.77, 0.92)	(0.45, 0.66)
College degree		0.51***	0.53***	0.21***
		(0.48, 0.55)	(0.49, 0.57)	(0.17, 0.26)
**1990s**				
High school or less		Ref.	Ref.	Ref.
Some college		0.84***	0.81***	0.39***
		(0.78, 0.91)	(0.73, 0.89)	(0.29, 0.52)
College degree		0.63***	0.73***	0.21***
		(0.58, 0.67)	(0.67, 0.80)	(0.15, 0.29)
**≥2000**				
High school or less		Ref.	Ref.	Ref.
Some college		1.01	1.05	0.64
		(0.90, 1.14)	(0.89, 1.23)	(0.32, 1.28)
College degree		1.11	1.19	0.31*
		(0.92, 1.33)	(0.94, 1.51)	(0.10, 0.97)

Note: Estimates were obtained using complex survey weights. 95% confidence intervals are in parenthesis. *** p < 0.001, ** p < 0.01, * p < 0.05.

Table S5. Relative risk ratios of CKMS stages for income by birth decade.

	**Base outcome**: Stage 0	**Outcome 1**: Stage 1	**Outcome 2**: Stage 2/3	**Outcome 3**: Stage 4
**≤1939**				
Low Income		Ref.	Ref.	Ref.
Middle Income		1.06	0.82	0.76
		(0.74, 1.53)	(0.63, 1.08)	(0.57, 1.01)
High Income		0.99	0.89	0.80
		(0.67, 1.45)	(0.66, 1.19)	(0.59, 1.08)
**1940s**				
Low Income		Ref.	Ref.	Ref.
Middle Income		1.02	0.92	0.75***
		(0.88, 1.18)	(0.82, 1.04)	(0.66, 0.84)
High Income		0.94	0.80***	0.59***
		(0.81, 1.10)	(0.71, 0.90)	(0.52, 0.67)
**1950s**				
Low Income		Ref.	Ref.	Ref.
Middle Income		0.96	0.86**	0.53***
		(0.84, 1.09)	(0.77, 0.95)	(0.47, 0.59)
High Income		0.90	0.71***	0.31***
		(0.80, 1.02)	(0.65, 0.78)	(0.28, 0.35)
**1960s**				
Low Income		Ref.	Ref.	Ref.
Middle Income		0.99	0.72***	0.33***
		(0.87, 1.12)	(0.64, 0.80)	(0.29, 0.38)
High Income		0.90	0.67***	0.23***
		(0.80, 1.02)	(0.61, 0.75)	(0.20, 0.26)
**1970s**				
Low Income		Ref.	Ref.	Ref.
Middle Income		0.74***	0.62***	0.21***
		(0.66, 0.83)	(0.55, 0.68)	(0.18, 0.25)
High Income		0.81***	0.73***	0.25***
		(0.72, 0.91)	(0.65, 0.81)	(0.21, 0.30)
**1980s**				
Low Income		Ref.	Ref.	Ref.
Middle Income		0.76***	0.71***	0.29***
		(0.70, 0.83)	(0.65, 0.77)	(0.24, 0.36)
High Income		0.66***	0.69***	0.20***
		(0.60, 0.72)	(0.63, 0.76)	(0.16, 0.25)
**1990s**				
Low Income		Ref.	Ref.	Ref.
Middle Income		1.00	1.03	0.48***
		(0.92, 1.08)	(0.93, 1.13)	(0.33, 0.71)
High Income		0.77***	0.94	0.36***
		(0.71, 0.83)	(0.85, 1.03)	(0.22, 0.58)
**≥2000**				
Low Income		Ref.	Ref.	Ref.
Middle Income		0.73***	0.87	0.44
		(0.63, 0.84)	(0.71, 1.07)	(0.19, 1.01)
High Income		0.73**	0.80	0.37
		(0.61, 0.89)	(0.62, 1.03)	(0.12, 1.13)

Note: Estimates were obtained using complex survey weights. 95% confidence intervals are in parenthesis. *** p < 0.001, ** p < 0.01, * p < 0.05.

Table S6. Relative risk ratios of CKMS stages for urban/rural residence by birth decade.

	**Base outcome**: Stage 0	**Outcome 1**: Stage 1	**Outcome 2**: Stage 2/3	**Outcome 3**: Stage 4
**≤1939**				
Urban		Ref.	Ref.	Ref.
Rural		1.01	1.06	1.23*
		(0.79, 1.29)	(0.88, 1.27)	(1.01, 1.50)
**1940s**				
Urban		Ref.	Ref.	Ref.
Rural		1.14*	1.09*	1.30***
		(1.03, 1.26)	(1.00, 1.18)	(1.18, 1.42)
**1950s**				
Urban		Ref.	Ref.	Ref.
Rural		1.06	1.06	1.31***
		(0.97, 1.15)	(0.99, 1.13)	(1.21, 1.41)
**1960s**				
Urban		Ref.	Ref.	Ref.
Rural		1.12**	1.22***	1.71***
		(1.04, 1.21)	(1.14, 1.31)	(1.57, 1.87)
**1970s**				
Urban		Ref.	Ref.	Ref.
Rural		1.25***	1.36***	1.98***
		(1.16, 1.35)	(1.27, 1.47)	(1.75, 2.23)
**1980s**				
Urban		Ref.	Ref.	Ref.
Rural		1.25***	1.34***	1.68***
		(1.17, 1.35)	(1.25, 1.45)	(1.41, 1.99)
**1990s**				
Urban		Ref.	Ref.	Ref.
Rural		1.40***	1.32***	1.56***
		(1.29, 1.51)	(1.20, 1.45)	(1.20, 2.03)
**≥2000**				
Urban		Ref.	Ref.	Ref.
Rural		1.25**	1.06	1.50
		(1.08, 1.44)	(0.89, 1.28)	(0.76, 2.97)

Note: Estimates were obtained using complex survey weights. 95% confidence intervals are in parenthesis. *** p < 0.001, ** p < 0.01, * p < 0.05.

Table S7. Distribution of age by birth decade

Age at time of interview	Birth Decade							
	**≤1939**	**1940s**	**1950s**	**1960s**	**1970s**	**1980s**	**1990s**	**≥2000**
18	0	0	0	0	0	0	0	4,722
19	0	0	0	0	0	0	0	4,879
20	0	0	0	0	0	0	2,149	2,996
21	0	0	0	0	0	0	2,503	3,352
22	0	0	0	0	0	0	4,003	1,989
23	0	0	0	0	0	0	4,371	2,068
24	0	0	0	0	0	0	6,489	120
25	0	0	0	0	0	0	7,257	0
26	0	0	0	0	0	0	7,088	0
27	0	0	0	0	0	0	7,563	0
28	0	0	0	0	0	0	8,266	0
29	0	0	0	0	0	0	8,159	0
30	0	0	0	0	0	3,283	6,500	0
31	0	0	0	0	0	2,972	5,733	0
32	0	0	0	0	0	6,613	3,504	0
33	0	0	0	0	0	6,719	3,407	0
34	0	0	0	0	0	9,894	204	0
35	0	0	0	0	0	11,445	0	0
36	0	0	0	0	0	11,018	0	0
37	0	0	0	0	0	11,440	0	0
38	0	0	0	0	0	12,403	0	0
39	0	0	0	0	0	11,858	0	0
40	0	0	0	0	4,199	9,864	0	0
41	0	0	0	0	3,588	8,006	0	0
42	0	0	0	0	8,512	4,905	0	0
43	0	0	0	0	8,215	4,532	0	0
44	0	0	0	0	11,641	236	0	0
45	0	0	0	0	13,259	0	0	0
46	0	0	0	0	12,425	0	0	0
47	0	0	0	0	13,519	0	0	0
48	0	0	0	0	13,683	0	0	0
49	0	0	0	0	13,917	0	0	0
50	0	0	0	5,785	11,731	0	0	0
51	0	0	0	4,666	9,867	0	0	0
52	0	0	0	11,108	6,077	0	0	0
53	0	0	0	11,160	5,877	0	0	0
54	0	0	0	16,525	318	0	0	0
55	0	0	0	18,658	0	0	0	0
56	0	0	0	17,448	0	0	0	0
57	0	0	0	18,303	0	0	0	0

58	0	0	0	19,523	0	0	0	0
59	0	0	0	19,517	0	0	0	0
60	0	0	7,683	14,969	0	0	0	0
61	0	0	6,866	12,841	0	0	0	0
62	0	0	15,387	7,809	0	0	0	0
63	0	0	14,819	7,088	0	0	0	0
64	0	0	21,940	431	0	0	0	0
65	0	0	25,194	0	0	0	0	0
66	0	0	23,118	0	0	0	0	0
67	0	0	23,627	0	0	0	0	0
68	0	0	23,324	0	0	0	0	0
69	0	0	22,061	0	0	0	0	0
70	0	8,206	16,495	0	0	0	0	0
71	0	7,367	13,883	0	0	0	0	0
72	0	15,385	8,149	0	0	0	0	0
73	0	13,186	7,249	0	0	0	0	0
74	0	18,920	378	0	0	0	0	0
75	0	19,051	0	0	0	0	0	0
76	0	17,155	0	0	0	0	0	0
77	0	15,670	0	0	0	0	0	0
78	0	14,290	0	0	0	0	0	0
79	0	12,824	0	0	0	0	0	0
80	29,294	58,654	0	0	0	0	0	0
Age range	80+	70-80	60-74	50-64	40-54	30-44	20-34	18-24

Table S8. Prevalence of CKMS stages by age group

	**CKMS stages**				Obs.
Age groups	Stage 0	Stage 1	Stage 2/3	Stage 4	
18-24	44.80	37.00	17.53	0.67	39,505
	(43.91, 45.69)	(36.15, 37.85)	(16.82, 18.25)	(0.53, 0.81)	
25-34	28.79	45.04	24.94	1.24	86,966
	(28.23, 29.34)	(44.42, 45.65)	(24.41, 25.47)	(1.11, 1.38)	
35-44	20.11	39.62	37.46	2.81	121,515
	(19.66, 20.56)	(39.06, 40.17)	(36.91, 38.01)	(2.58, 3.04)	
45-54	14.57	29.17	49.85	6.41	149,479
	(14.18, 14.96)	(28.67, 29.68)	(49.30, 50.39)	(6.15, 6.67)	
55-64	10.94	18.86	58.68	11.52	202,448
	(10.64, 11.25)	(18.46, 19.25)	(58.19, 59.17)	(11.20, 11.84)	
65-74	8.48	11.58	63.19	16.74	225,342
	(8.22, 8.75)	(11.28, 11.89)	(62.73, 63.65)	(16.40, 17.09)	
75+	8.48	8.06	58.97	24.49	164,866
	(8.17, 8.79)	(7.77, 8.36)	(58.41, 59.54)	(24.00, 24.98)	

Note: Estimates were obtained using complex survey weights. 95% confidence intervals are in parenthesis.
